# Construction of an immune-related gene signature for the prognosis and diagnosis of glioblastoma multiforme

**DOI:** 10.3389/fonc.2022.938679

**Published:** 2022-08-02

**Authors:** Ziye Yu, Huan Yang, Kun Song, Pengfei Fu, Jingjing Shen, Ming Xu, Hongzhi Xu

**Affiliations:** ^1^ Department of Neurosurgery, Huashan Hospital, Shanghai Medical College, Fudan University, Shanghai, China; ^2^ National Center for Neurological Disorders Huashan Hospital, Shanghai Medical College, Fudan University, Shanghai, China; ^3^ Shanghai Key Laboratory of Brain Function and Restoration and Neural Regeneration, Huashan Hospital, Shanghai Medical College, Fudan University, Shanghai, China; ^4^ Neurosurgical Institute of Fudan University, Huashan Hospital, Shanghai Medical College, Fudan University, Shanghai, China; ^5^ Shanghai Clinical Medical Center of Neurosurgery, Huashan Hospital, Shanghai Medical College, Fudan University, Shanghai, China; ^6^ Department of Nursing, Huashan Hospital, Fudan University, Shanghai, China; ^7^ Department of Anesthesiology, Huashan Hospital, Fudan University, Shanghai, China

**Keywords:** glioblastoma multiforme, immune-related gene, immune, prognosis, bioinformatic analysis

## Abstract

**Background:**

Increasing evidence has suggested that inflammation is related to tumorigenesis and tumor progression. However, the roles of immune-related genes in the occurrence, development, and prognosis of glioblastoma multiforme (GBM) remain to be studied.

**Methods:**

The GBM-related RNA sequencing (RNA-seq), survival, and clinical data were acquired from The Cancer Genome Atlas (TCGA), Genotype-Tissue Expression (GTEx), Chinese Glioma Genome Atlas (CGGA), and Gene Expression Omnibus (GEO) databases. Immune-related genes were obtained from the Molecular Signatures Database (MSigDB). Differently expressed immune-related genes (DE-IRGs) between GBM and normal samples were identified. Prognostic genes associated with GBM were selected by Kaplan–Meier survival analysis, Least Absolute Shrinkage and Selection Operator (LASSO)-penalized Cox regression analysis, and multivariate Cox analysis. An immune-related gene signature was developed and validated in TCGA and CGGA databases separately. The Gene Ontology (GO) and Kyoto Encyclopedia of Genes and Genomes (KEGG) analyses were performed to explore biological functions of the signature. The correlation between immune cell infiltration and the signature was analyzed by single-sample gene set enrichment analysis (ssGSEA), and the diagnostic value was investigated. The gene set enrichment analysis (GSEA) was performed to explore the potential function of the signature genes in GBM, and the protein–protein interaction (PPI) network was constructed.

**Results:**

Three DE-IRGs [Pentraxin 3 (*PTX3*), *TNFSF9*, and bone morphogenetic protein 2 (*BMP2*)] were used to construct an immune-related gene signature. Receiver operating characteristic (ROC) curves and Cox analyses confirmed that the 3-gene-based prognostic signature was a good independent prognostic factor for GBM patients. We found that the signature was mainly involved in immune-related biological processes and pathways, and multiple immune cells were disordered between the high- and low-risk groups. GSEA suggested that *PTX3* and *TNFSF9* were mainly correlated with interleukin (IL)-17 signaling pathway, nuclear factor kappa B (NF-κB) signaling pathway, tumor necrosis factor (TNF) signaling pathway, and Toll-like receptor signaling pathway, and the PPI network indicated that they could interact directly or indirectly with inflammatory pathway proteins. Quantitative real-time PCR (qRT-PCR) indicated that the three genes were significantly different between target tissues.

**Conclusion:**

The signature with three immune-related genes might be an independent prognostic factor for GBM patients and could be associated with the immune cell infiltration of GBM patients.

## Introduction

Glioblastoma multiforme (GBM) is the most common and aggressive primary brain tumor, accounting for about 50% of all gliomas (1). The highly invasive nature and relapse rate prevent long-term survival despite surgical removal, radiation, chemotherapy, and targeted therapy (2, 3). After current standard therapy, the mean overall survival (OS) of GBM patients was 14.6 months, 2-year survival rate was 26.5%, and 5-year survival rate was about 5% (4, 5).

Increasing evidence has demonstrated that inflammation is a relevant marker to promote tumorigenesis and progression (6). Inflammation can advance the proliferation and survival of tumor cells (7) and improve the blood circulation of the tumor (8). Immune-related genes are widely studied in the field of inflammatory diseases such as osteoarthritis (9), and some researchers also have deeply explored the correlation between immune-related genes and kidney renal clear cell carcinoma (10), breast cancer (11), and pancreatic cancer (12). However, the roles of immune-related genes in the occurrence, development, and prognosis of GBM remain to be researched. The immune microenvironment is composed of glioma-associated immune cells, such as microglia, macrophages, and B cells, and immunoregulatory factors, such as interleukin (IL)-6, IL-10, and transforming growth factor β (TGF-β), which regulate the progression of glioma (13). The immune microenvironment is intimately connected to the emergence, invasion, and metastasis of tumor and plays a critical role in tumor diagnosis, prevention, and prognosis (14, 15). As a new approach to cancer treatment, the development of immunotherapy brings new dawn to GBM patients. However, there are limited results in the current research and application of GBM (16). Therefore, it is imperative for effective clinical decision-making to develop GBM prognostic biomarkers and establish a prognostic model.

Bioinformatic methods were used in our study to explore the immune-related genes in GBM, establish a prognostic model, and obtain deeper insight in the relationship and interaction pathways between GBM and the immune microenvironment, which can generate inspiration for the early diagnosis, prognosis improvement, and development of new therapeutic targets.

## Materials and methods

### Data sources

GBM-related RNA sequencing (RNA-seq) (with high count value) and clinical data of 157 GBM patients, including 148 GBM patients with survival information and nine GBM patients without survival information, and five matched normal brain samples were acquired from The Cancer Genome Atlas (TCGA) (https://portal.gdc.cancer.gov/repository) database. Moreover, we acquired the RNA-seq (with high count value) of 209 normal brain samples from the Genotype-Tissue Expression (GTEx) database. The Chinese Glioma Genome Atlas (CGGA) dataset with complete survival information was downloaded and used as the validation set, including 237 GBM samples. GSE4290 dataset, including 77 GBM samples and 23 normal samples, was downloaded and used to screen differentially expressed genes (DEGs). Immune-related gene sets [Hallmark gene set and Gene Ontology (GO) annotation] were acquired from the Molecular Signatures Database (MSigDB) (17–19) and merged to obtain immune-related genes.

### Identification of differentially expressed immune-related genes

The batch effect used “sva” R package between TCGA and GTEx (20). Next, DEGs between normal brain samples and GBM samples of TCGA and GTEx databases were screened using the “limma” package of R software with the following criteria: |log2 fold-change (FC)| >1 and *P* < 0.05 (21, 22). Similarly, DEGs between normal brain samples and GBM samples from the GSE4290 dataset were obtained. Furthermore, differentially expressed immune-related genes (DE-IRGs) were obtained by taking the intersection of the DEGs obtained from TCGA and GTEx, the DEGs of the GSE4290 dataset, and the immune-related genes.

### Evaluation of the differentially expressed immune-related gene model

According to the median expression of the DE-IRGs, the patients in TCGA database were divided into a target gene high-expression group (n = 74) and a target gene low-expression group (n = 74). First, the DE-IRGs associated with prognosis were screened using Kaplan–Meier survival analysis with *P*-value <0.05. Second, Least Absolute Shrinkage and Selection Operator LASSO-penalized Cox regression analysis *via* the “glmnet” R package (23) was used to filter false-positive genes. Finally, multivariate Cox analysis was used to retain the model genes.

The 148 GBM samples with complete survival information in TCGA and GTEx databases were divided into a training set and a test set according to 1:1, and 237 GBM samples with complete survival information in the CGGA dataset were used as the validation set. According to the median risk score, GBM patients were separated into the high- and low-risk groups. The risk score was calculated as follows: risk score = ∑βgene_(i)_ ×Exp gene_(i)_ (1=1-n) in which β represents regression coefficient.

### Independent prognostic analysis and development of a predictive nomogram

Based on the sample of 148 TCGA patients with complete clinical information, we incorporated the risk model, age, gender, and other clinicopathological factors into the risk model for univariate Cox independent prognostic analysis. Then, we included the clinicopathological factors into the multivariate Cox analysis. Additionally, we generated a nomogram to predict the survival years for the GBM patients using the “rms” (https://CRAN.R-project.org/package=rms) and “survival” packages (24). Lastly, Harrell’s concordance index (C-index) and calibration curves were employed to evaluate the prediction accuracy of the nomogram (25).

### Gene ontology functional and Kyoto encyclopedia of genes and genomes pathway enrichment analysis

The DEGs between the high- and low-risk groups were selected by DESeq2 (|log2(FC)| ≥1, *P* ≤ 0.05); then, GO and KEGG analysis was conducted with the “clusterProfiler” of R package (26). The infiltrating scores of 24 immune cells and the immune-related pathways were calculated with single-sample gene set enrichment analysis (ssGSEA) (27) in the high- and low-risk groups. According to the results of the ssGSEA, the differences in immune cell infiltration between high- and low-risk groups were analyzed.

### Identification of the diagnostic value of prognostic genes

Receiver operating characteristic (ROC) curves were then employed to investigate the model’s predictive validity, and the area under the curve (AUC) was calculated with TCGA dataset and GSE4290 dataset using the “survivalROC” package in R software (28, 29). The “GGploT2” R package was used to compare the differences among Pentraxin 3 (*PTX3*), *TNFSF9*, and bone morphogenetic protein 2 (*BMP2*) genes in clinical factors O6-methylguanine-DNA methyltransferase (MGMT) methylation, age, gender, *1p/19q* codeletion, and isocitrate dehydrogenase (*IDH*) mutation.

### Single-sample gene set enrichment analysis of signature genes

The ssGSEA was conducted based on the gene list sorted by Spearman correlation coefficient between the specified signature gene and every gene of TCGA dataset to explore the significant biological processes and pathways associated with the signature gene.

### Construction of protein–protein interaction network

The protein–protein interaction (PPI) network was constructed by the Search Tool for the Retrieval of Interacting Genes (STRING, version: 11.0, https://string-db.org) database (30) to recognize its potential interaction relationships at the protein level between the model genes and immune-related pathway genes. A confidence >0.6 was included in the PPI networks. The PPI network was visualized by Cytoscape 3.8.0 software (http://www.cytoscape.org/index.html) (31).

### Clinical tissue collection

All validation samples were collected with the consent of the patient, and ethical permission was obtained from Huashan Hospital Affiliated to Fudan University. During 2021, seven postoperative clinical specimens from five adult male patients with glioblastoma were collected from the Department of Neurosurgery in our hospital, including five tumor tissue samples and two adjacent tissue samples.

### Quantitative real-time PCR

Total RNA was extracted by TRIzol reagent (cat.: 356281) in five GBM samples (CA) and two normal samples. The Synthesis All-in-OneTM First-Strand cDNA Synthesis Kit (cat.: G33330-500) was used to synthesize the first-strand cDNA. Quantitative PCR was performed using 2× Universal Blue SYBR Green qPCR Master Mix (cat.: G3326-05). The primers included the following: BMP2-F: GTTTTGATGTCACCCCCGCT, BMP2-R: TCCAGTCATTCCACCCCACG; PTX3-F: CTATTTTATTCCCAATGCGTT, PTX3-R: CCAGTTTGTTCTCCTCTCCAC; TNFSF9-F: TGTTCTGCTGATCGATGGG, TNFSF9-R: CAGTGTGAAGATGGACGCC; GAPDH-F: CCCATCACCATCTTCCAGG, GAPDH-R: CATCACGCCACAGTTTCCC. The relative mRNA expression data were calculated with the 2^−ΔΔCt^ method.

### Statistical analysis

All statistical analyses were conducted in the R programming language and environment (version 4.0.3). Kaplan–Meier curves were generated by R package “survminer” (version 0.4.9), *P*-values were calculated by log-rank tests. Univariate and multivariate Cox regression analyses were conducted to analyze the related factors affecting the OS of GBM patients. Spearman rank correlation was acquired to analyze the correlations between the DEGs and infiltrating immune cells. *P* < 0.05 was set as criterion.

## Results

### Identification of differentially expressed genes and differentially expressed immune-related genes

**Figure 1:**
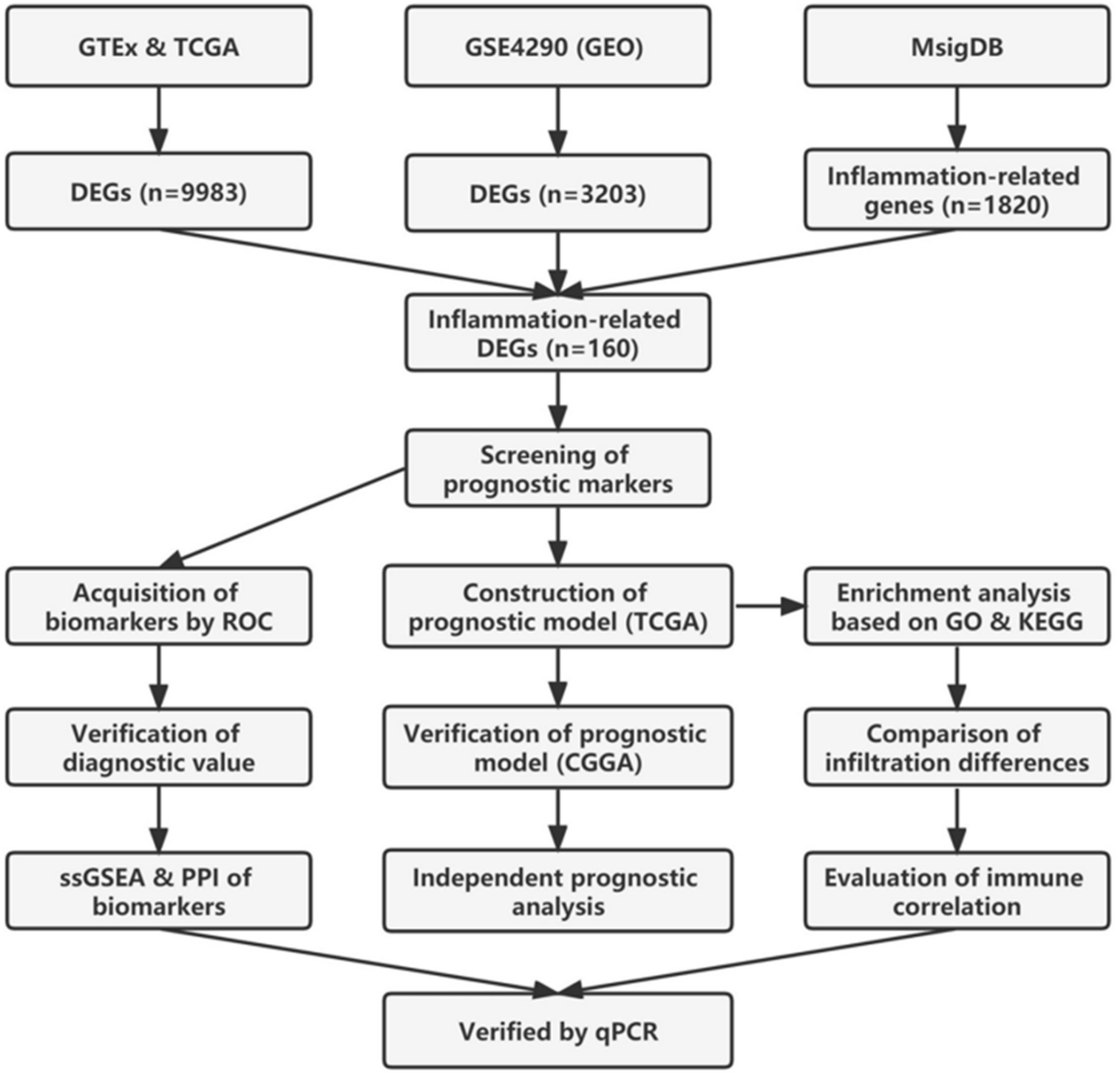
Flowchart of the present study.

**Figure 2:**
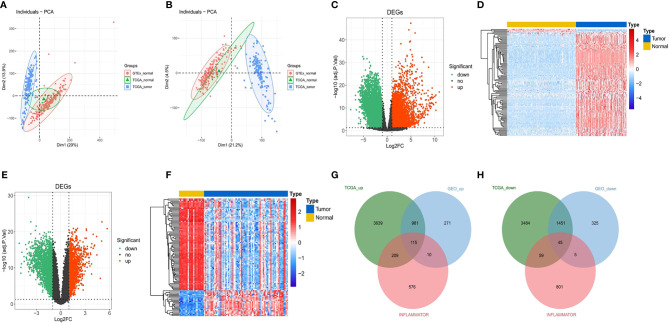
Identification of DEGs and DE-IRGs. **(A)** Primordial principal component analysis (PCA) of all samples. **(B)** PCA of all samples after batch correction. **(C)** Volcano plot for DEGs between TCGA and GTEx. The transverse reference line represents -log10(adjust. *P*-value) = 0.05, and the longitudinal reference line represents log2FC = ± 1. **(D)** Heat map (Top100) for DEGs between TCGA and GTEx. Blue indicates low expression, and red indicates high expression. **(E, F)** Volcano plot and heat map (Top100) for DEGs in the GSE4290 dataset. **(G, H)** Upregulated immune-related genes and downregulated immune-related genes after matching the DEGs from the above analyses.

The study flowchart of this article is shown in **Figure 1**. TCGA and GTEx data were merged to remove batch effects by analysis of the DESeq2 package (32). After batch effect correction, batch differences between GTEx and TCGA normal samples became relatively small, while between-group differences between GTEx normal samples and TCGA tumor samples became larger (**Figures 2A, B** **)**.

We obtained 214 normal samples and 157 tumor samples from TCGA and GTEx. Then, a total of 9,983 DEGs between 214 normal samples and 157 tumor samples, including 4,944 upregulated DEGs and 5,039 downregulated DEGs, were screened (**Figures 2C, D** **)**. In the differential expression analysis in the GSE4290 gene set, including 23 normal samples and 77 tumor samples, 3,203 genes were found to be differentially expressed, among which 1,377 were upregulated and 1,826 were downregulated (**Figures 2E, F** **)**. Finally, a total of 160 DE-IRGs were significantly different between the tumor samples and normal samples in the DEG analysis for the Gene Expression Omnibus (GEO) cohort and TCGA cohort, including 115 upregulated DE-IRGs (**Figure 2G**) and 45 downregulated DE-IRGs (**Figure 2H**).

### Identification of prognostic differentially expressed immune-related genes

**Figure 3:**
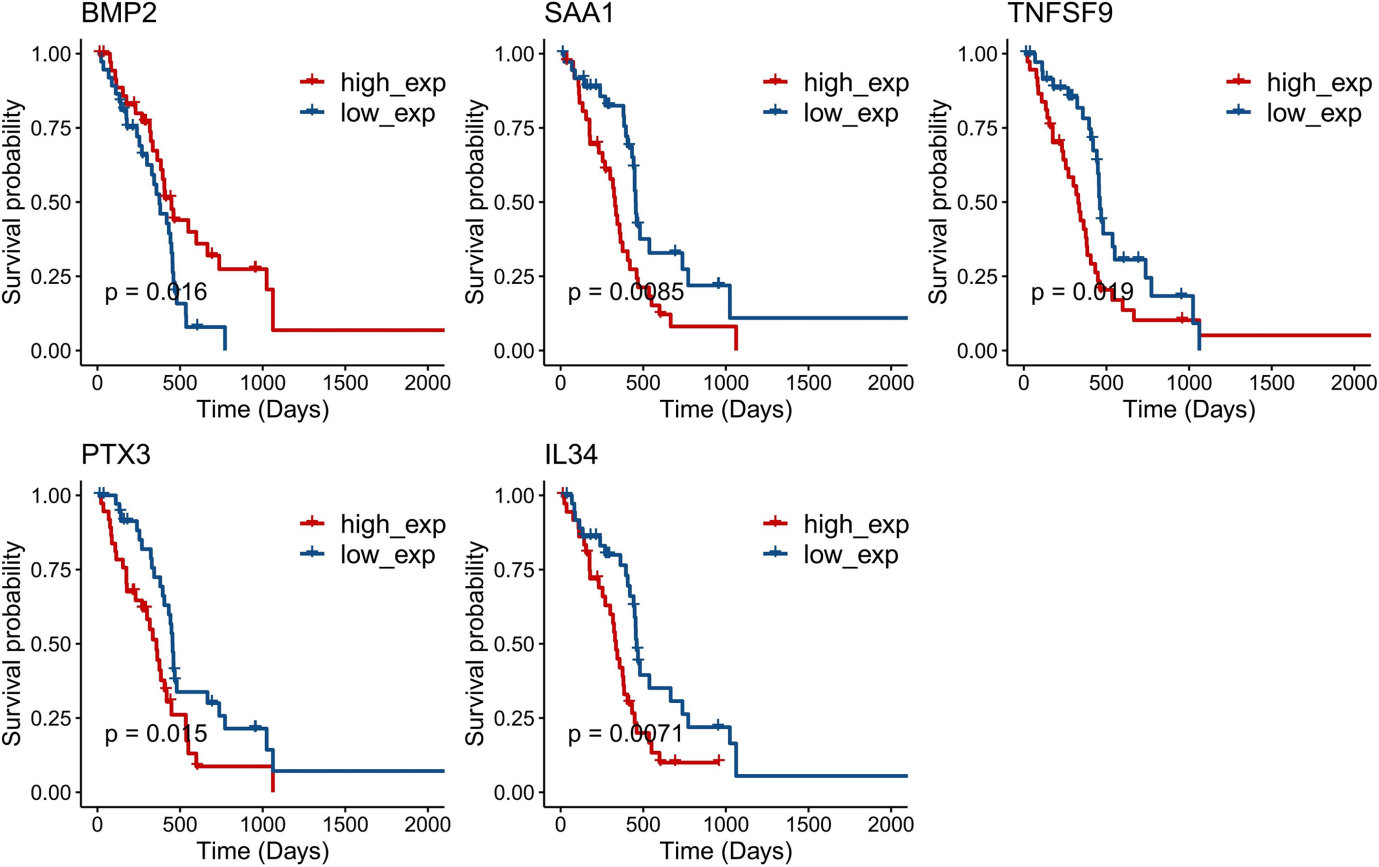
Kaplan–Meier survival curves of five single genes (*P* < 0.05): *IL34* (*P* = 0.0071), *SAA1* (*P* = 0.0085), *PTX3* (*P* = 0.015), *BMP2* (*P* = 0.016), and *TNFSF9* (*P* = 0.019).

**Figure 4:**
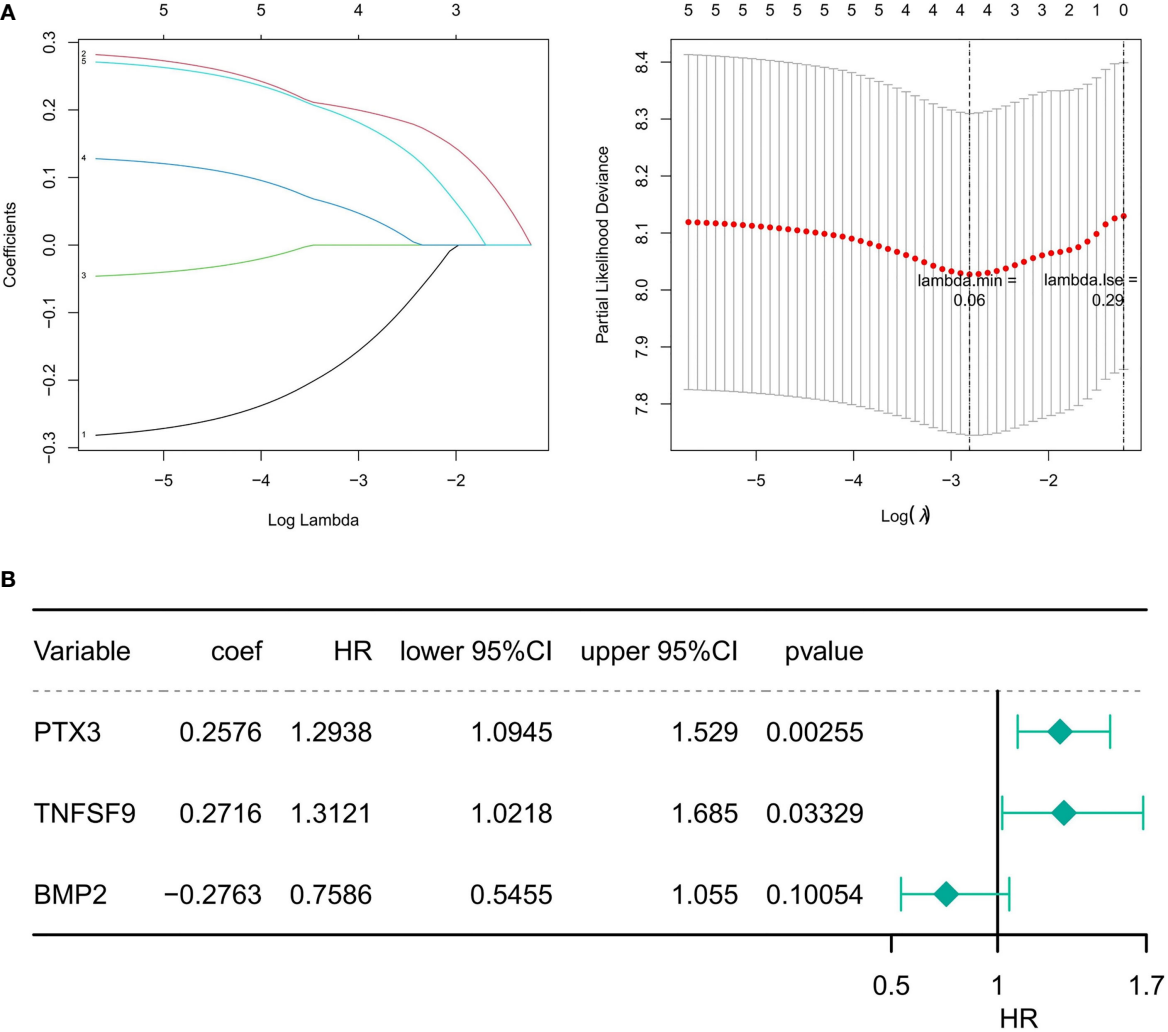
Identification of prognostic DE-IRGs. **(A)** The diagram of gene coefficient and the error diagram of cross validation in LASSO Cox regression. **(B)** Forest plot of the risk model constructed by the multivariate Cox regression analysis. HR is the hazard ratio, and lower/upper 95% CI is the 95% confidence interval of the risk value.

A total of five genes were identified as prognosis-associated genes by Kaplan–Meier survival analysis: IL34 (*P* = 0.0071), *SAA1* (*P* = 0.0085), *PTX3* (*P* = 0.015), *BMP2* (*P* = 0.016), and *TNFSF9* (*P* = 0.019) (**Figure 3**). Moreover, LASSO Cox regression identified four genes (*IL34, PTX3, BMP2, TNFSF9*) with the lambda = 0.06 (**Figure 4A**). Finally, *PTX3, BMP2, and TNFSF9* were selected and used to establish a DE-IRG signature based on their expression in the regression coefficient acquired from the multivariate Cox regression analysis (**Figure 4B**). Namely, the risk score of each patient was calculated according to the following formula: risk score = ∑βgene_(i)_ × Exp gene_(i)_ (1=1-n)= 0.2576 ×Exp *PTX3* +0.2716 × Exp *BMP2* + (-0.2763) ×Exp *TNFSF9*.

### Evaluation of the differentially expressed immune-related gene signature-based risk model in the training set

**Figure 5:**
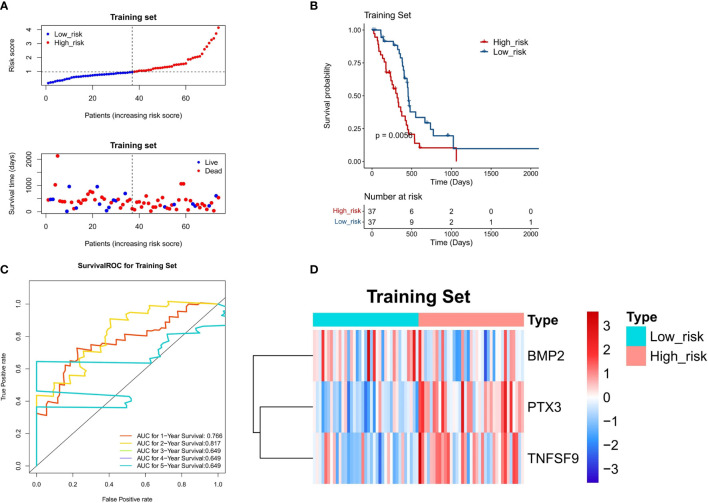
Evaluation of the DE-IRG signature-based risk model in the training set (n = 74). **(A)** The prognostic curve and a scatter plot for the training set. **(B)** The Kaplan–Meier OS curves of the high- and low-risk groups for the training set (*P* = 0.0056). **(C)** ROC curve for the training set (AUC >0.6). **(D)** The heat map of the gene expression profiles of the three model genes in the training set.

The three DE-IRGs were utilized to establish a DE-IRG signature. Based on the median value of the risk score, the patients with GBM were stratified into high- or low-risk group (**Figure 5A**). The risk score and the survival status of each patient were shown in the prognostic curve and a scatter plot, respectively (**Figure 5A**). We can see that the death cases were mainly distributed in the high-risk group from the scatter plot (**Figure 5A**). The Kaplan–Meier OS curves of the two groups were significantly different, which show that the high-risk group has a poorer prognosis than that of the low-risk group (*P* = 0.0056; **Figure 5B**). The area under the curve (AUC) values of a time‐dependent ROC curve for 1-, 2-, 3-, 4-, and 5-year OS were 0.766, 0.817, 0.649, 0.649, and 0.649, respectively (**Figure 5C**), and the 3-gene-based risk model had considerable prognostic predictive validity. The gene expression profiles of the three genes were shown in the heat map (**Figure 5D**).

### Validation of a 3-gene-based prognostic model using the test set and Chinese glioma genome atlas dataset

**Figure 6:**
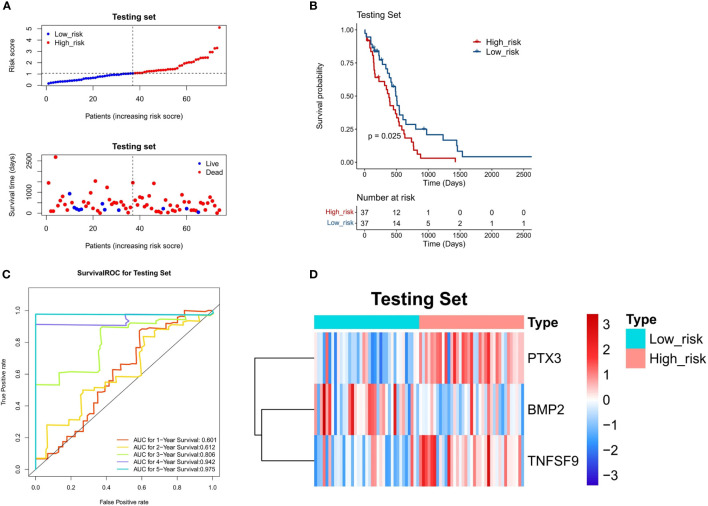
Validation of a 3-gene-based prognostic model using the test set (n = 74). **(A)** The prognostic curve and a scatter plot for the test set. **(B)** The Kaplan–Meier OS curves of the high- and low-risk groups for the test set (*P* = 0.025). **(C)** ROC curve for the test set (AUC >0.6). **(D)** The heat map of the gene expression profiles of the three model genes in the test set.

**Figure 7:**
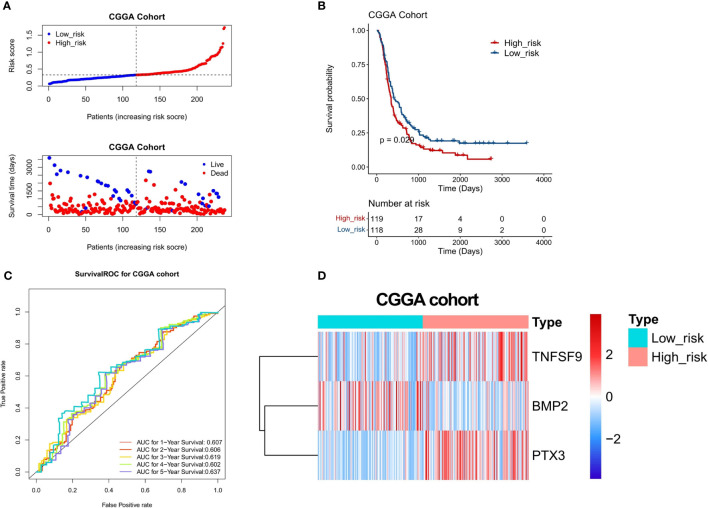
Validation of a 3-gene-based prognostic model using the CGGA validation set (n = 248). **(A)** The prognostic curve and a scatter plot for the validation set. **(B)** The Kaplan–Meier OS curves of the high- and low-risk groups for the validation set (*P* = 0.029). **(C)** ROC curve for the validation set (AUC >0.6). **(D)** The heat map of the gene expression profiles of the three model genes in the validation set.

To confirm the stability of the 3-gene-based prognostic model, we then used it to predict OS in the test set (n = 74) and CGGA dataset (n = 248) using the median risk score as the cutoff. As shown in **Figures 6A** and **7A**, the test set and CGGA dataset were classified into a low-risk group and a high-risk group, from which the scatter plot indicated that the live cases were mainly distributed in the low-risk group. The Kaplan–Meier OS curves of the test set (*P* = 0.025) and CGGA dataset (*P* = 0.029) were shown to have good prognoses in both high- and low-risk groups. The AUC values of a time‐dependent ROC curve for 1-, 2-, 3-, 4-, and 5-year OS were higher than 0.6 (**Figures 6C**, **7C**). The heat map showed that the gene expression profiles of the three genes have a similar tendency. Combining the results from the training set, test set, and CGGA dataset, the 3-gene-based prognostic model built in this study had satisfactory specificity and sensitivity.

### Independent prognostic analysis of the risk score

**Figure 8:**
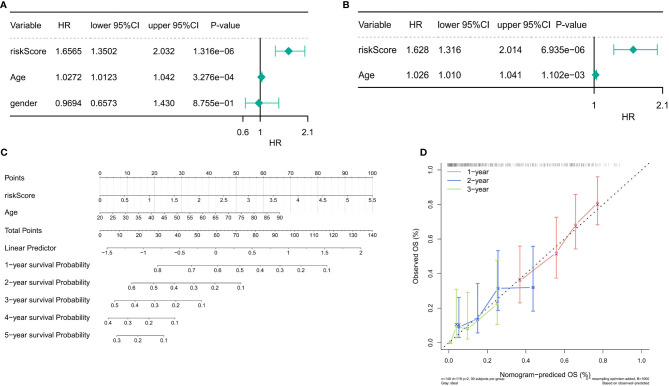
Independent prognostic analysis of the risk score. **(A)** Univariate Cox regression analysis in the training set. **(B)** Multivariate Cox regression analysis in the training set (*P* < 0.05). **(C)** The nomogram designed with the risk score model and age (C-index = 0.66). **(D)** The 1–3-year calibration curve.

Univariate and multivariate Cox regression analyses were performed to evaluate the prognostic significance of the 3-gene-based prognostic model combined with clinicopathologic parameters. In the sample of 148 TCGA patients with complete clinical information, univariate Cox regression analyses indicated that the *P*-values of the risk score (*P* = 1.316e-06) and age (*P* = 3.276e-04) were <0.05 (**Figure 8A**). In addition, multivariate Cox regression analysis indicated that the risk score (*P* = 6.935e-06) and age (*P* = 1.102e-03) were independent prognostic factors (**Figure 8B**). Next, the nomogram was designed with the risk score model and age. Furthermore, the C-index of the nomogram was 0.66, indicating that the nomogram model had a certain predictive value (**Figure 8C**). Because the number of samples with a survival period of more than 4 and 5 years was too few to draw the corresponding calibration curve, the calibration curve results for only 1–3 years were presented in **Figure 8D**, which exposed that the survival rate obtained by the model was nearly equal to the actual survival rate.

### Diagnostic value of model genes

**Figure 9:**
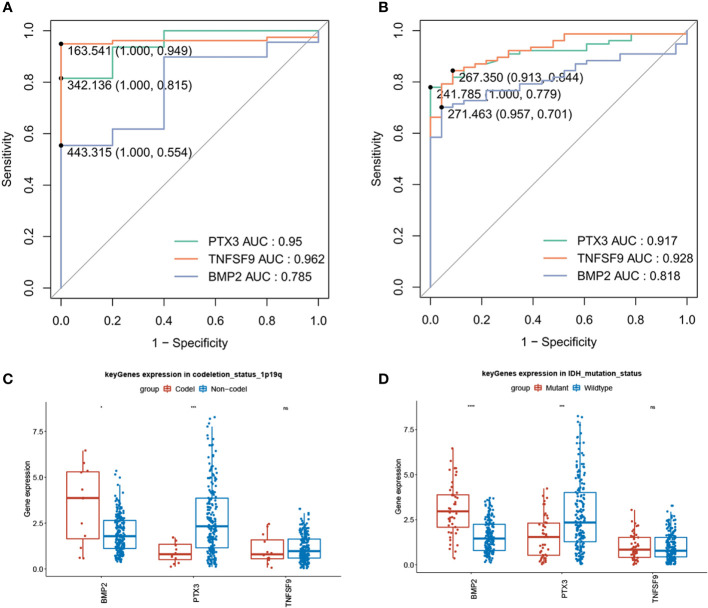
Diagnostic value of model genes (AUC >0.7) and significant differences in gene *BMP2* and *PTX3* between 1p/19q codeletion and IDH mutation (*P* < 0.05). **(A)** Single-gene ROC curve for the three model genes in TCGA dataset. **(B)** Single-gene ROC curve for the three model genes in the GSE4290 dataset. **(C)** Compared with *1p/19q* non-codeletion, BMP2 was high expression in *1p/19q* codeletion, while *PTX3* was low expression. **(D)** In contrast with the IDH wild type, *BMP2* showed a strong expression in *IDH* mutation, while *PTX3* showed a weak expression.

We plotted single-gene ROC curves for the three model genes in TCGA dataset and the GSE4290 dataset, respectively, as shown in **Figures 9A, B**. The AUC values of the three model genes are more than 0.7, indicating a satisfactory accuracy of prediction. Meanwhile, there was no significant difference in the expression of the three genes in clinical factors such as MGMT methylation, age, and gender, while there were significant differences in gene *BMP2* and *PTX3* between *1p/19q* codeletion and *IDH* mutation (**Figures 9C, D** **)**.

### Correlation analysis of immunity and inflammation in high- and low-risk groups

**Figure 10:**
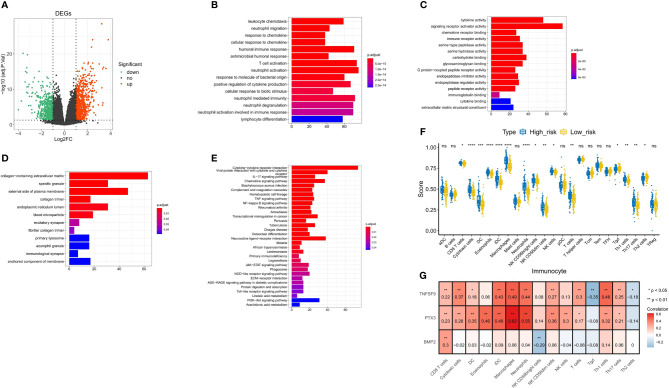
Correlation analysis of immunity and inflammation in the high- and low-risk groups. **(A)** Volcano plot for DEGs between the high- and low-risk groups. **(B–D)** The top 15 terms enriched by GO-BP/MF/CC functional enrichment analyses. **(E)** Thirty-two pathways enriched by KEGG functional enrichment analysis. **(F)** Difference of 15 immune cell infiltrations between the high- and low-risk groups. **(G)** Spearman correlation between three model genes and 15 differential immune cells. * p<0.05 vs. control; ** p<0.01 vs. control; *** p<0.001 vs. control; **** p<0.0001 vs. control; ns, not statistically significant.

We obtained 1,563 DEGs in the high- and low-risk groups of the training test, including 862 upregulated DEGs and 701 downregulated DEGs (**Figure 10A**). Next, the results of GO analysis of DEG signature showed that these DEGs were significantly enriched in 251 biological process (BP) terms, 37 molecular function (MF) terms, and 12 cellular component (CC) terms. The top enriched GO terms for GO-BP terms were neutrophil activation, T-cell activation, neutrophil-mediated immunity, etc.; for GO-MF terms were cytokine activity and signaling receptor activator activity; and for GO-CC terms were collagen-containing extracellular matrix and external side of plasma membrane. The top 15 GO-BP/MF/CC terms were visualized in **Figures 10B-D**. In the results of KEGG analysis, we found 32 significantly enriched KEGG pathways that were presented in **Figure 10E**, and the cytokine–cytokine receptor interaction pathway was most enriched.

To further explore the correlation between the risk score and immune status, we quantified the enrichment scores of diverse immune cell subpopulations and related functions or pathways with ssGSEA. We screened 15 immune cells for differences in infiltration between the high- and low-risk groups (**Figures 10F, G** **)**. Then, we calculated Spearman correlations of three model genes with 15 differential immune cells. As shown in **Figure 10**, *TNFSF9* was positively correlated with CD8 T cells (r = 0.22, *P* < 0.01), cytotoxic cells (r = 0.37, *P* < 0.01), dendritic cells (DCs) (r = 0.18, *P* < 0.05), immature dendritic cells (iDCs) (r = 0.43, *P* < 0.01), macrophages (r = 0.49, *P* < 0.01), neutrophils (r = 0.44, *P* < 0.01), natural killer (NK) CD56dim cells (r = 0.27, *P* < 0.01), T cells (r = 0.3, *P* < 0.01), Tgd (r = -0.35, *P* < 0.01), Th1 cells (r = 0.46, *P* < 0.01), Th17 cells (r = 0.25, *P* < 0.01), and Th2 cells (r = -0.18, *P* < 0.05). *PTX3* was positively correlated with CD8 T cell (r = 0.23, *P* < 0.01), cytotoxic cells (r = 0.28, *P* < 0.01), DCs (r = 0.35, *P* < 0.01), eosinophils (r = 0.46, *P* < 0.01), iDCs (r = 0.48, *P* < 0.01), macrophages (r = 0.62, *P* < 0.01), neutrophils (r = 0.55, *P* < 0.01), NK CD56dim cells (r = 0.36, *P* < 0.01), NK cells (r = 0.3, *P* < 0.01), T cells (r = 0.17, *P* < 0.05), Th1 cells (r = 0.32, *P* < 0.01), and Th17 cells (r = 0.21, *P* < 0.05). *BMP2* was positively correlated with CD8 T cell (r = 0.3, *P* < 0.01) and NK CD56bright cells (r = 0.36, *P* < 0.01).

**Figure 11:**
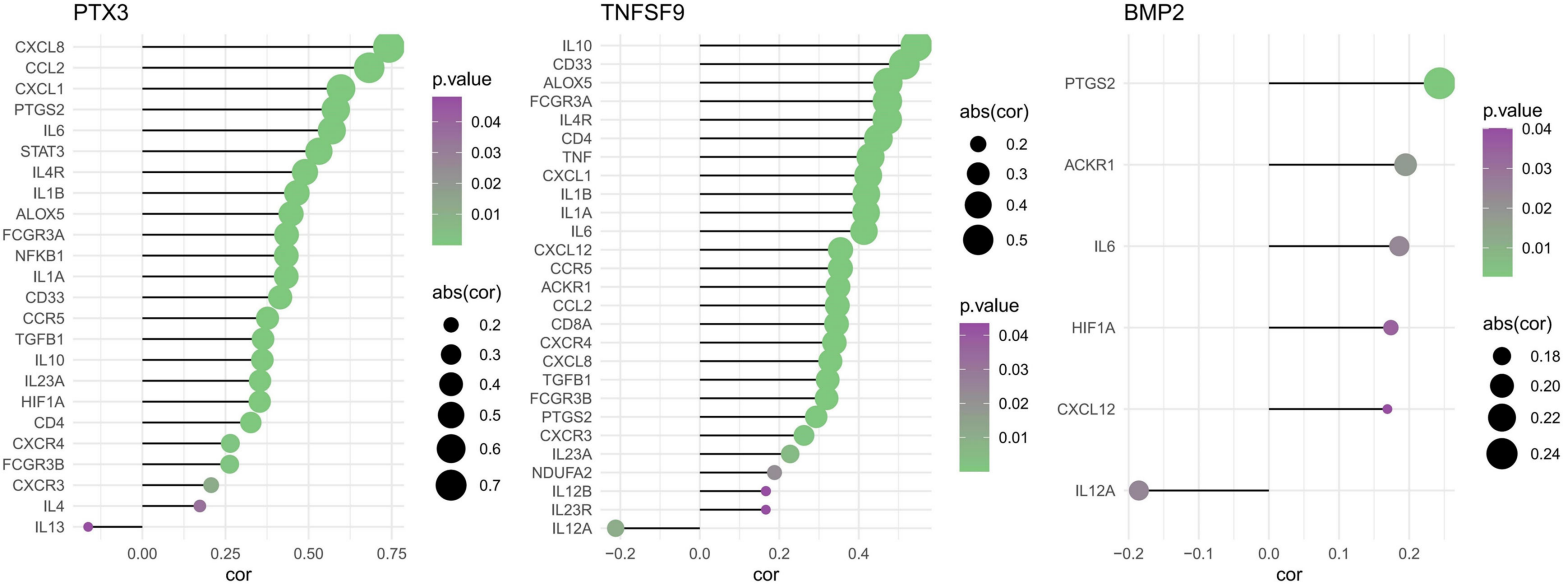
Spearman correlations of three model genes and inflammatory factors (*P* < 0.05).

Spearman correlations of three model genes and inflammatory factors were also calculated, showing results with *P*-values <0.05 (**Figure 11**). The *PTX3* was positively correlated with CXCL8, CCL2, CXCL1, PTGS2, IL6, STAT3, IL4R, IL1B, ALOX5, FCGR3A, NFKB1, IL1A, CD33, CCR5, TGFB1, IL10, IL23A, HIF1A, CD4, CXCR4, FCGR3B, CXCR3, IL4, and IL13. The *TNFSF9* was positively correlated with IL10, CD33, ALOX5, FCGR3A, IL4R, CD4, TNF, CXCL1, IL1B, IL1A, IL6, CXCL12, CCR5, ACKR1, CCL2, CD8A, CXCR4, CXCL8, TGFB1, FCGR3B, PTGS2, CXCR3, IL23A, NDUFA2, IL12B, IL23R, and IL12A. The *BMP2* was just positively correlated with PTGS2, ACKR1, IL6, HIF1A, CXCL12, and IL12A.

### Single-sample gene set enrichment analysis of model genes

**Figure 12:**
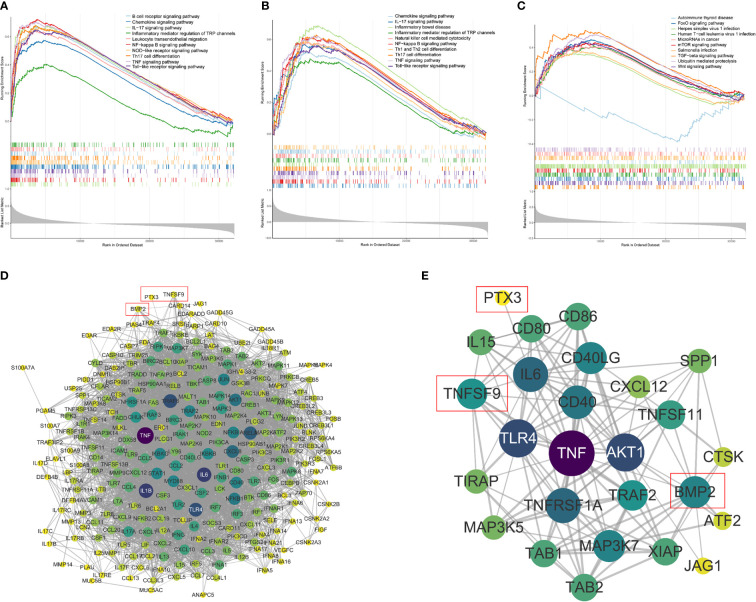
Single-sample gene set enrichment analysis (ssGSEA). **(A–C)** The ssGSEA of PTX3, TNFSF, and BMP2, including gene enrichment scores, hit and ranked list metric. **(D)** The PPI network between the model genes and the four genes directly related to inflammatory factor pathways (confidence = 0.6). **(E)** The PPI subnetwork related to PTX3, TNFSF9, and BMP2.

Furthermore, to explore the molecular functions underlying the GBM, gene set enrichment analysis (GSEA) was used to analyze the possible functional pathways of the three model genes. In the ssGSEA of *PTX3*, 196 pathways were enriched, including B-cell receptor signaling pathway, Chemokine signaling pathway, IL−17 signaling pathway, and Inflammatory mediator regulation of TRP channels. In the ssGSEA of *TNFSF9*, 164 pathways were enriched, mainly enriched in Chemokine signaling pathway, IL−17 signaling pathway, Inflammatory bowel disease, and other pathways. We found that the four genes directly related to inflammatory factor pathways of *PTX3* were enriched the same as the *TNFSF9*, which included the IL-17 signaling pathway, NF-κB signaling pathway, TNF signaling pathway, and Toll-like receptor signaling pathway (**Figures 12A, B** **)**. In the ssGSEA of *BMP2*, 54 pathways were enriched. We selected 10 pathways related to immunity and various diseases and cancers for display (**Figure 12C**).

A gene interaction network was constructed to illustrate the relationships between the model genes and the four genes directly related to inflammatory factor pathways (**Figure 12D**) that included 274 nodes and 4,526 edges. The gene interaction networks related to *PTX3*, TNFSF9, and *BMP2* were extracted to draw subnetworks (**Figure 12E**), which were composed of 26 nodes and 126 edges. It can also be seen that compared with the other two genes, BMP2 was connected to more nodes.

### Expression level of model genes in target tissues

**Figure 13:**
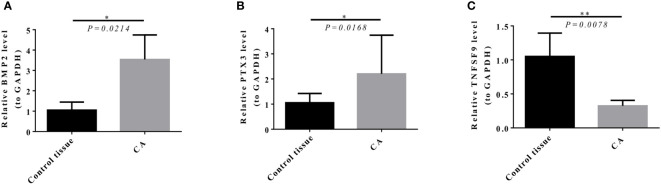
Expression level of model genes in target tissues verified by qRT-PCR (the average value of three repeated experiments). **(A, B)** BMP2 (*P* = 0.0214) and PTX3 (*P* = 0.0168) expression in GBM was significantly higher than that in control tissue. **(C)** TNFSF9 (*P* = 0.0078) expression in GBM was significantly lower than that in control tissue.

The results of qRT-PCR showed that *BMP2* (*P* = 0.0214) and *PTX3* (*P* = 0.0168) expression in GBM was significantly higher than in paracancerous tissue (**Figures 13A, B** **)**, and *TNFSF9* (*P* = 0.0078) expression in GBM was significantly lower than in paracancerous tissue (**Figure 13C**).

## Discussion

Immune inflammatory response plays an important role in the occurrence and proliferation of tumor cells, angiogenesis in tumor tissues, and invasion and metastasis of cancer cells. Among them, innate immune cell infiltration and the production and aggregation of inflammatory chemokines are typical manifestations of the tumor-related inflammatory response. Immune inflammatory response can activate a series of molecular biological signaling pathways bound up with tumor cell generation, proliferation, and metastasis (33–35). As part of the tumor environment, the inflammatory microenvironment is also correlated with tumor occurrence. Inflammatory cytokines in the tumor focus and blood circulation may be necessary for the proliferation and metastasis of tumor cells (36). Studies had shown that the immune inflammatory response was involved in the formation of glioma (37).

At present, many types of prognostic signatures have been constructed for GBM (38, 39). Based on data from TCGA and CGGA, Fan et al. (40) identified 426 DEGs after comparing global gene expression patterns in GBM samples and controls. Upon univariate and LASSO regression analyses, seven DEGs were considered of prognostic value, i.e., *CLEC5A, HOXC6, HOXA5, CCL2, GPRASP1, BSCL2*, and *PTX3* (40). Based on demographic and clinical measures, prognostic nutritional index and neutrophil/lymphocyte ratio have been identified to be independent prognostic factors for GBM patients (39). Tewarie et al. (41) had summarized and analyzed the research related to establishing GBM prognosis models from 2010 to 2019. Despite the increasing development of survival prediction models for GBM patients, only seven models have been validated retrospectively in an external patient cohort (42–48), and none has been validated prospectively (41). Gittleman et al. (47) built a nomogram using the Cox proportional hazards model and identified the factors that increased the probability of shorter survival that included advanced age, male gender, lower Karnofsky performance score (KPS), subtotal resection, and unmethylated MGMT status. This model has been deployed as a publicly available prediction tool. However, due to the complexity of GBM, no model has been applied as a standardized tool to guide clinical decision-making, which leaves sufficient space for further research. This study thoroughly explored the factors related to the prognosis of GBM for immune-related genes and conducted rigorous validation group analysis using the datasets from different sources. The prognostic model was solidly verified according to the results of qRT-PCR analysis of clinical samples. Compared with the existing studies, this article focuses on the construction of a GBM prognosis model in the direction of immune-related genes, which may not consider the comprehensiveness of the prognosis model. However, it did not ignore the external patient cohort verification and biological verification, which provided a reliable theoretical basis for further prospective research. Inflammation is closely related to tumorigenesis and development (6); emerging treatment methods of GBM such as immunotherapy are mainly guided by the inflammatory microenvironment mechanism (16). The DE-IRGs provided in our article may pioneer new thinking of GBM therapeutics.

In previous studies, *PTX3*, a member of the pentraxin superfamily, is rapidly produced by multiple cell types in response to primary inflammatory signals (49, 50). The high expression of *PTX3* may be regulated by JNK-Jun, IKK/nuclear factor kappa B (NF-κB), and Wnt signaling pathways, so as to promote the expression of epithelial–mesenchymal transition (EMT)-related proteins and enhance the migration and invasion abilities of tumor cells (51). As a key molecule of bone metabolism, *PTX3* overexpression can affect osteoclast differentiation and promote bone metastasis of breast cancer and gastric cancer (52). *PTX3* may promote the stemness of tumor cells through Hedgehog and Hippo-YAP signaling pathways, so as to expedite tumor growth and malignant progression (53). The overexpression of PTX3 is a poor prognosis sign in lots of cancer types such as lung cancer (54), cervical cancer (55), colorectal cancer (56), pancreatic cancer (57), breast cancer (58), gastric cancer (59), melanoma (60), and squamous cell carcinoma of the head and neck (61). However, *PTX3* also has the inhibitory effect on angiogenesis and is able to moderate malignant progression in bladder cancer (52), multiple myeloma (62), fibrosarcoma (63), and prostate cancer (64). When it comes to GBM, PTX3 can promote the proliferation and metastasis of glioma cells, which has been found to indicate a terrible prognosis (65).

*TNFSF9* (CD137L), the counterreceptor for CD137 (4-1BB) and a member of the tumor necrosis factor (TNF) ligand superfamily (66), can be expressed on the surface of antigen-presenting cells (APCs) as a transmembrane protein, and the stimulation can be transmitted to APCs through reverse TNFSF9 signal (67). *TNFSF9* signal plays a role in activating and secreting pro-inflammatory cytokines in monocytes and inhibiting the release of anti-inflammatory cytokines such as IL-10 (68). In addition, *TNFSF9* signal has the ability to induce the activation, migration, survival, and differentiation of monocytes (69) and has also been proven to participate in NK cell-mediated antitumor immunity (70). The studies found that *TNFSF9* facilitates antitumor immunity in liver cancer (66) and inhibited the proliferation of small cell lung cancer cells and induced apoptosis (71). However, Wu et al. (72, 73) discovered that *TNFSF9* promotes the metastasis of pancreatic cancer through Wnt/Snail signal transduction and regulates M2 polarization of macrophages through Src/FAK/p-Akt/IL-1β signal transduction. In the field of glioma, research focusing on *TNFSF9* is rare, and only a few studies ended up just at the stage of clinical data analysis. Mu et al. (74) reported that GBM patients with a high expression of *TNFSF9* had a longer OS, but Cui et al. (75) showed that there was no significant correlation between the level of TNFSF9 and GBM patient survival.

*BMP2*, bone morphogenetic protein 2, belongs to the transforming growth factor β (TGF-β) superfamily (76). They play an important role in the growth and development of the body by coordinating the differentiation, proliferation, and apoptosis of cells in different tissues and organs (77). Many studies have revealed that BMPs not only regulate bone and cartilage but also exert a variety of biological processes in the development of cancers (78), including breast cancer (79), ovarian cancer (80), and lung cancer bone metastasis (81). The expression level of BMP2 is related to the degree of tumor malignancy and GBM patient survival; therefore, it is being considered as a prognostic marker for glioma (82, 83). BMP2 increased the differentiation and apoptosis of glioma in a concentration-dependent manner (84) through the downregulation of both MGMT and hypoxia-inducible factor-1 (HIF-1) (xref>/xref>). In addition, *BMP2* has also been reported to render glioblastoma stem-like cells more susceptible to temozolomide treatment through destabilization of HIF-1 (82, 85).

In our study, 15 immune cells were screened for differences in infiltration between high- and low-risk groups and were confirmed to be closely associated with model genes. For instance, *TNFSF9* and *PTX3* were mostly correlated with macrophages, and *BMP2* was mostly relevant to CD8 T cells. The rise of macrophages may represent a negative feedback from downregulated immune cells. Tumor-associated macrophages have been reported to contribute to a poor prognosis of GBM patients (38, 86), which is consistent with the fact that macrophages increased in the high-risk group in our article. CD8 T cells are the principal force to eliminate glioma cells, but they are easily exhausted and cannot be effectively supplemented, accounting for a low proportion in the GBM immune microenvironment (87). NK cells are one type of immune cells recruited first to the glioma area. They can secrete perforin and granzyme to induce apoptosis or necrosis of target cells, without limitation from major histocompatibility complex (MHC) (88). The function of NK cells was inhibited, especially in high-grade glioma (89). Myeloid-derived suppressor cells (MDSCs) are heterogeneous cells, including immature macrophages, granulocytes, and iDCs. There is abundant infiltration of MDSCs in glioma tissue. Its phagocytosis decreases, and the expression of immunosuppressive molecules IL-10, TGF-β, and B7H1 increases, so as to inhibit the differentiation of DCs, reduce the cytotoxicity of NK cells, and induce T-cell apoptosis (90).

To consummate our research, the possible functional pathways of the three model genes were excavated. IL-17, produced by Th17 cells, was demonstrated to promote tumor development through the induction of a tumor-promoting microenvironment at tumor sites (91). Cui et al. (75) reported a direct correlation between progression-free survival and low incidence of IL-17-producing cells, suggesting that the presence of IL-17-producing cells may be a good prognostic marker for gliomas. NF-κB transcription factor and NF-κB pathway are overexpressed in leukemia, gastrointestinal tumors, especially in glioma cells, suggesting the correlation between the development of glioma and various NF-κB-mediated immune responses. When NF-κB signal is abnormal, especially overexpression, it can accelerate the division cycle of tumor cells and disorder the immune regulation function, leading to tumor immune evasion (92). Taking immune cells and immune factors as nodes, immune-related pathways connect them in a series and weave the complex inflammatory response network around GBM, which together form a complex tumor immune microenvironment.

The treatment of recurrent and progressive GBM is still a challenging problem in oncology. In recent years, immunotherapy has achieved great success in the treatment of malignant tumors, and many attempts have been implemented to the experimental and even clinical treatment of glioma (93). Preclinical studies have shown that blocking programmed cell death protein-1 (PD-1) or cytotoxic T lymphocyte-associated antigen-4 (CTLA-4) can significantly inhibit the growth of glioma cells and prolong the survival time of experimental animals (94). Targeting glioma-specific antigens such as EGFRvIII, IL13Rα2, and HER2 also showed anti-glioma effects in mouse models (95). So far, these immunotherapies have not been proven to be effective in large-scale phase III clinical trials (96), which may be attributed to blood–brain barrier, tumor heterogeneity, and glioma inhibitory immune microenvironment (97).

In order to further develop immunotherapy for GBM, our research explored immune-related genes, which can participate in the construction of the GBM prognosis model, and analyzed the related immune cells and immune signaling pathways across the board. The comprehensiveness of our study is still insufficient, and the patient information such as chemotherapy and radiotherapy is not included in the establishment of the prognosis model, which is due to the lack of relevant information in the datasets. Due to the limitations of objective conditions, the conclusions have not been adequately verified by *in vivo* or *in vitro* experiments, and the foundation for further mechanism research remains to be consolidated. Similar to other GBM prognostic models, this model still lacks prospective evidence, which reminds us of the necessity of continuing to pay attention to the research advances on these model genes in designing and implementing clinical prospective research.

## Conclusion

The signature with three immune-related genes (*PTX3*, *TNFSF9*, and *BMP2*) might be an independent prognostic factor of GBM patients and could be associated with the immune cell infiltration of GBM patients.

## Data availability statement

The datasets presented in this study can be found in online repositories. The names of the repository/repositories and accession number(s) can be found in the article/supplementary material.

## Ethics statement

The studies involving human participants were reviewed and approved by The Institutional Review Board of Huashan Hospital, Fudan University. The patients/participants provided their written informed consent to participate in this study.

## Author contributions

ZY and HX conceived the project. ZY and HY obtained the requisite data, conducted the statistical analysis, interpreted the data, drafted the manuscript, generated tables and figures, and received the final manuscript submission. KS, PF, JS, and MX critically revised the manuscript draft. All authors contributed to the article and approved the submitted version.

## Acknowledgments

The authors sincerely thank all the patients who participated in this study.

## Conflict of interest

The authors declare that the research was conducted in the absence of any commercial or financial relationships that could be construed as a potential conflict of interest.

## Publisher’s note

All claims expressed in this article are solely those of the authors and do not necessarily represent those of their affiliated organizations, or those of the publisher, the editors and the reviewers. Any product that may be evaluated in this article, or claim that may be made by its manufacturer, is not guaranteed or endorsed by the publisher.
